# Theoretical Studies on Catalysis Mechanisms of Serum Paraoxonase 1 and Phosphotriesterase Diisopropyl Fluorophosphatase Suggest the Alteration of Substrate Preference from Paraoxonase to DFP

**DOI:** 10.3390/molecules23071660

**Published:** 2018-07-07

**Authors:** Hao Zhang, Ling Yang, Ying-Ying Ma, Chaoyuan Zhu, Shenghsien Lin, Rong-Zhen Liao

**Affiliations:** 1 College of Life Science and Engineering, Northwest Minzu University, Lanzhou 730030, China; zhang_hao@mail.bnu.edu.cn; 2 MIIT Key Laboratory of Critical Materials Technology for New Energy Conversion and Storage, School of Chemistry and Chemical Engineering, Harbin Institute of Technology, Harbin 150001, China; 3 Inner Mongolia University of Technology, Hohhot 010051, China; yyma@imut.edu.cn; 4 Department of Applied Chemistry, Institute of Molecular Science and Center for Interdisciplinary Molecular Science, National Chiao-Tung University, Hsinchu 30050, Taiwan; cyzhu@mail.nctu.edu.tw (C.Z.); sheng@mail.nctu.edu.tw (S.L.); 5 Key Laboratory for Large-Format Battery Materials and System, Ministry of Education School of Chemistry and Chemical Engineering, Huazhong University of Science and Technology, Wuhan 430074, China; rongzhen@hust.edu.cn

**Keywords:** enzyme evolution, hydrolysis, β-propeller protein, plasticity, reaction mechanism

## Abstract

The calcium-dependent β-propeller proteins mammalian serum paraoxonase 1 (PON1) and phosphotriesterase diisopropyl fluorophosphatase (DFPase) catalyze the hydrolysis of organophosphorus compounds and enhance hydrolysis of various nerve agents. In the present work, the phosphotriesterase activity development between PON1 and DFPase was investigated by using the hybrid density functional theory method B3LYP. Based on the active-site difference between PON1 and DFPase, both the wild type and the mutant (a water molecule replacing Asn270 in PON1) models were designed. The results indicated that the substitution of a water molecule for Asn270 in PON1 had little effect on the enzyme activity in kinetics, while being more efficient in thermodynamics, which is essential for DFP hydrolysis. Structure comparisons of evolutionarily related enzymes show that the mutation of Asn270 leads to the catalytic Ca^2+^ ion indirectly connecting the buried structural Ca^2+^ ion via hydrogen bonds in DFPase. It can reduce the plasticity of enzymatic structure, and possibly change the substrate preference from paraoxon to DFP, which implies an evolutionary transition from mono- to dinuclear catalytic centers. Our studies shed light on the investigation of enzyme catalysis mechanism from an evolutionary perspective.

## 1. Introduction

Studies based on structural comparisons of evolutionally related enzymes can not only identify the role of the catalysis related residues, but also aid in understanding the way followed by the evolution of enzymes for new catalytic functions [1,2,3,4,5,6,7]. Recently, such studies have received more attention for the general insights into the enzymatic function. After reporting the evolution of the phosphotriesterase activity in the metallo-β-lactamase family [8], in the present work, we will investigate phosphotriesterase activity development in other evolutionally related enzymes, as well as the co-evolution of the active-site structure and catalytic function. The phosphotriesterase activity is highly focused for their detoxification ability toward agricultural pesticides, by degrading organophosphate (OP) compounds [9,10,11].

The mammalian serum paraoxonase 1 (PON1) is a calcium-dependent serum esterase which catalyzes the hydrolysis of a broad range of organic esters and OP compounds [12]. Although PON1 is a lactonase with native substrates γ- and *δ*-lactones which have long alkyl side chains [13,14,15], it is more fascinating that PON1 possesses promiscuous OP hydrolase activity, particularly on paraoxon, which is attributed to its considerable plasticity of catalytic structure [4,15]. The crystal structures show that PON1 has a six-blade β-propeller fold with two calcium ions in its central tunnel [15,16]. The structural Ca^2+^ is completely embedded inside the protein, and the catalytic Ca^2+^ is located at the bottom of the active site cavity.

The squid phosphotriesterase diisopropyl fluorophosphatase (DFPase) from *Loligo vulgaris* [17,18,19], another structurally related calcium containing β-propeller protein, shows relatively specific substrate preference, efficiently catalyzing the hydrolysis of diisopropyl fluorophosphate (DFP) and G-type nerve agents, including tabun (GA), sarin (GB), soman (GD), and cyclohexyl sarin (GF). The detoxification of the OP agent is achieved by the hydrolytic reaction producing a phosphate or phosphonate and a fluoride ion [19,20]. Since both DFPase and PON1 display distinct detoxication by catalyzing OP hydrolysis, they were well studied as catalytic bioscavengers to degrade OP compounds.

PON1 and DFPase have been always compared together, for their structural similarity and functional homology [4,16]. DFPase, isolated from the central nervous system of the squid, shows strong preference for the hydrolysis of P–F or P–CN bonds, which are absent in natural compounds [21,22,23]. PON1’s native function is likely to be a lactonase which hydrolyzes the lactones from the oxidized lipids [24,25], but its promiscuous hydrolase activity on OP received more attention [4,15,16]. Thus, the common detoxifying function of both enzyme systems via OP hydrolysis was focused upon here.

**Figure 1:**
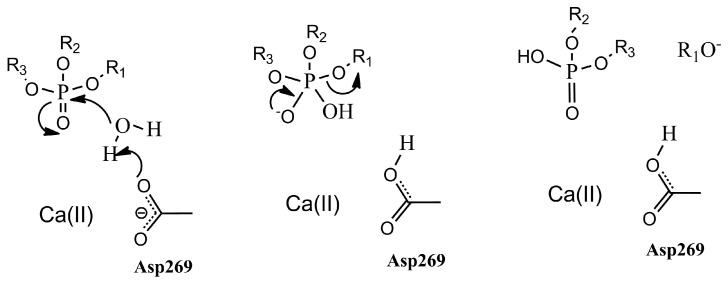
The reaction mechanism for PON1 and DFPase.

It was suggested that PON1 and DFPase employ similar catalytic mechanisms as phosphotriesterase, due to their structural similarities of active sites [15,26]. The attacking nucleophile for phosphotriester hydrolysis was identified, by our recent work, to be an activated water molecule, with the nucleophile attacking the phosphorus center (Figure 1) [27]. This conclusion was supported by the experimental research of Ben-David Moshe et al.: that the E53Q and D269N mutants in PON1 both possessed measurable lactonase and paraoxonase activity, and sufficient mutation studies combined with related molecular dynamics simulations suggested that the water activated by Glu53 and Asp269 should be the most likely attacking nucleophile [4]. Since, in our previous work, we have fully discussed the chemical property details of the catalytic mechanism [27], in the present work, we will mainly focus on the rate-determining reaction step of the OP hydrolysis catalyzed both by DFPase and PON1, and investigate the biological significance of active-site residues. 

**Figure 2:**
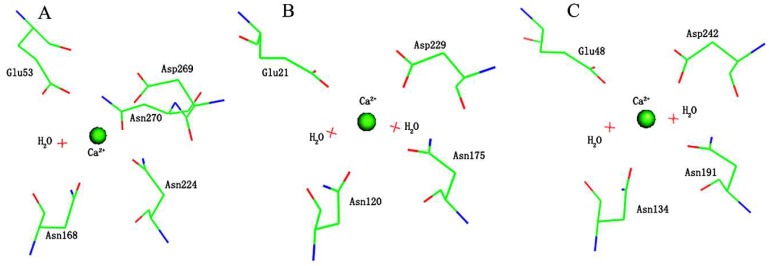
Active site structures of PON1 (PDB code: 3SRE) [15] (**A**), DFPase (PDB code: 2GVW) [18] (**B**) and XC5397 (PDB code: 3DR2) [28] (**C**), respectively. These figures are drawn by the PyMOL [44].

The crystal structures of several structurally related enzymes PON1, DFPase, and the gluconolactonase from *Xanthomonas campestris* (XC5397) [28] adopt a classical six-blade β-propeller fold, while the coordinated ligand Asn270 of the metal center is observed in PON1, exclusively (Figure 2A–C). Among all the coordinating residues in PON1, the role of Asn270 has always been ignored. The site-specific saturation mutagenesis indicated Asn270 seems to play the smallest role in catalysis for both paraoxon and thio-buthyl butyriclactone (TBBL) [4]. However, from an evolutionary perspective, the mutation rate at active sites, such a core functional region, should be very low across a protein family. Once such mutations have occurred, they should lead to new functional branches in a protein family [29,30,31]. Thus Asn270, preserved in PON1 exclusively, among the family with the typical six-blade β-propeller fold, should have a certain evolutionary meaning for the core enzymatic function. Thus, in this study, we investigated the role of Asn270 for the catalytic function of PON1 and the potential reason for it preserving in PON1, but being absent in DFPase, by using hybrid density functional theory (DFT) with B3LYP functional combined with the cluster models of the active site. The methods were widely and successfully used on the enzyme reaction modeling by Ramos’s group of zinc enzymes [32,33,34,35], Himo and Siegbahn’s group [36,37,38,39], and Russo et al. [40,41,42,43].

Although the evolution of the enzymatic structures and mechanisms have been mentioned early [45,46], so far, most studies still focused on the catalytic mechanism of an individual enzyme, which provides limited information on the enzymatic function. In this study, in order to explore the development of the phosphotriesterase activity between PON1 and DFPase, we have made a more comprehensive study on the catalytic mechanisms of the evolutionally related enzymes. The knowledge of how nature has evolved new catalytic reactions via the conserved structural features at active sites can help understand the diversity of the enzyme catalytic mechanisms and further help guide enzyme design in the laboratory [5,6].

## 2. Results and Discussion

### 2.1. Effects of Asn270 Mutation on Catalytic Reaction

**Figure 3:**
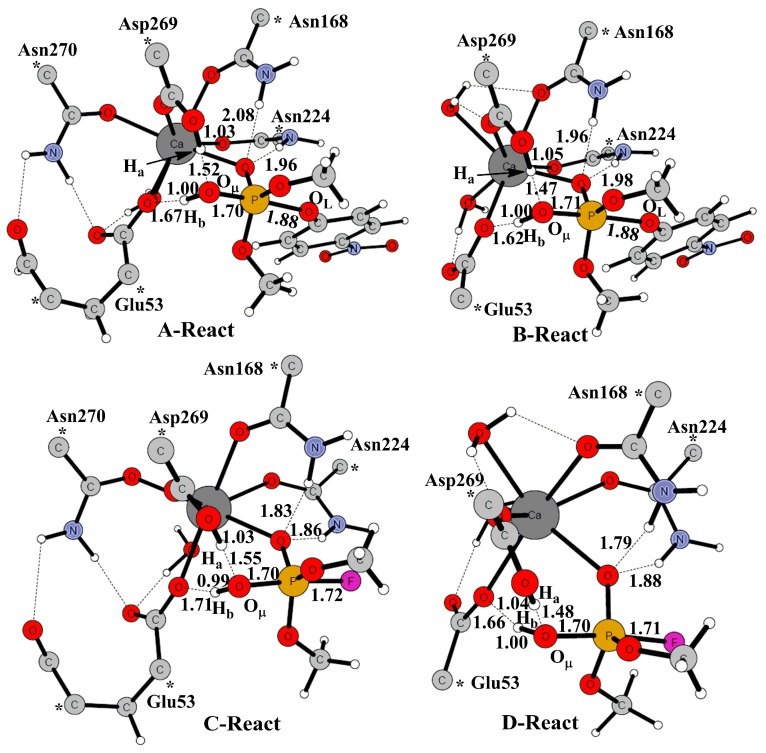
Optimized geometries of reactants along the reaction paths of (**A**) PON1W-Paraoxon, (**B**) PON1M-Paraoxon, (**C**) PON1W-DFP, and (**D**) PON1M-DFP models. The frozen atoms are marked with asterisks.

The active-site models of wild type PON1 (Active-site-W) were built on the basis of the high-resolution crystal structure (PDB code: 3SRE) [15]. Then, the models of the catalysis mechanism via the attacking of a water molecule (named W1 here) were designed by adding the substrates paraoxon and DFP into the Active-site-W model, called PON1W-Paraoxon model and PON1W-DFP model, respectively. These models contain one Ca^2+^ ion and the coordinating residues (Asp269, Glu53, Asn168, Asn224, and Asn270) (Figure 3, A-React and C-React). These residue ligands were truncated, and the side chains were retained. In order to keep the optimized structures close to the crystal structures, the truncation atoms were frozen at their X-ray positions in the geometry optimization. The frozen atoms are marked with asterisks. Hydrogen atoms were added manually, and the truncated bonds were saturated by hydrogen atoms. This procedure has been widely applied in the studies on reaction mechanisms [11,47,48,49]. The total charge of models is zero. 

Furthermore, in order to investigate the effects of Asn270 on the phosphotriesterase activity, the mutant models, Active-site-M, PON1M-Paraoxon, and PON1M-DFP models, were derived from the Active-site-W, PON1W-Paraoxon, and PON1W-DFP models, respectively, by replacing Asn270 into a water molecule (named W2 here) (Figure 3, B-React and D-React). These mutant models have the same coordination as in DFPase (Figure 2), which also represent the active-site models of DFPase. In addition, the main-chain oxygen atom of Glu53, makes a stable hydrogen bond with the side-chain nitrogen atom of Asn270. Thus the main-chain oxygen atom of Glu 53 and the hydrogen bond with the side-chain nitrogen atom of Asn270 were considered in the wild type models (PON1W-Paraoxon and PON1W-DFP), while absent in the mutant models (PON1M-Paraoxon and PON1M-DFP) for the mutation of Asn270. 

**Figure 4:**
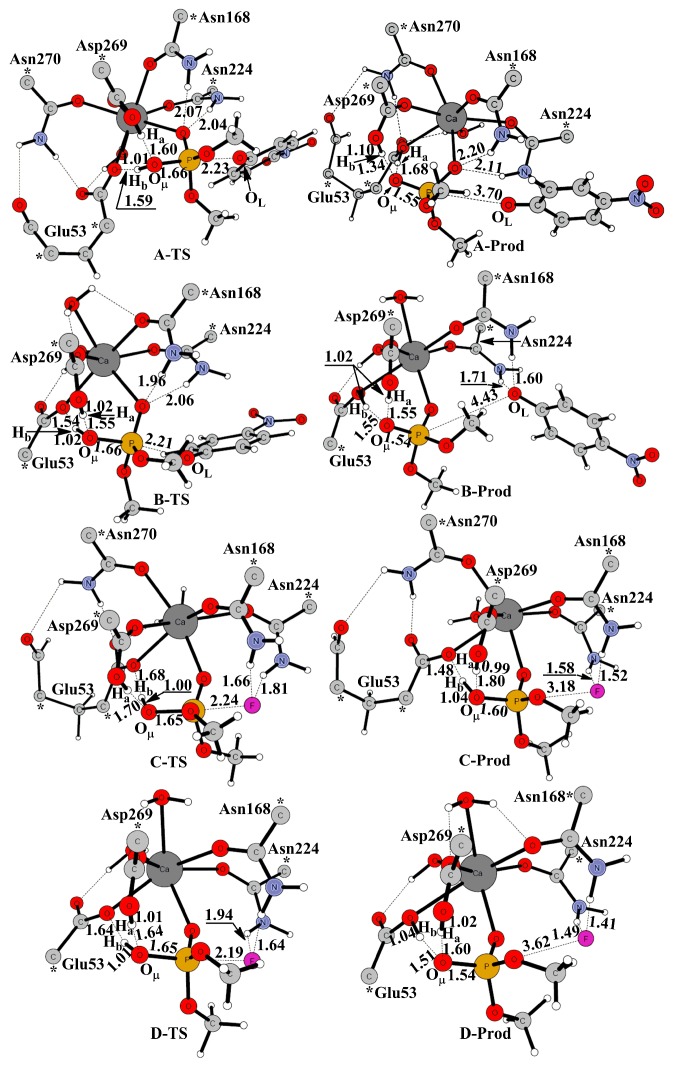
Optimized geometries of transition states and products along the reaction paths of (**A**) PON1W-Paraoxon, (**B**) PON1M-Paraoxon, (**C**) PON1W-DFP, and (**D**) PON1M-DFP models. The frozen atoms are marked with asterisks.

The reaction is supposed to proceed by a two-step addition–elimination (An + Dn) mechanism [50], which goes through a common pentavalent intermediate observed in phosphoryl transfer reactions. Similar to the mechanism of DFPase [27], the first step is the activation of H_2_O by Asp269 and Glu53 to form a nucleophile hydroxide and a metastable pentavalent complex. Here, we mainly focused on the Dn step. Optimized geometries of the models active-site-W, active-site-M, and the four Michaelis complexes (A–D-React in Figure 3) PON1W-Paraoxon, PON1M-Paraoxon, PON1W-DFP, and PON1M-DFP were listed in Table 1. From Table 1, we can see that either replacing Asn270 or adding substrates results in almost consistent changes of the coordination bonds. Bond lengths of Ca–O_Glu53_ and Ca–O_Asn168_ decreased, while the Ca–O_Asp269_ and Ca–O_Asn224_ bonds increased. For the mutant models, the substitute water molecule W2 connects the side chain of Asp269 via a strong hydrogen bond, and the hydrogen bond distances of O_W2_bO_Asp269_ are ca. 2.6–2.7 Å along the reaction paths (see Figure 3 and Figure 4), which is consistent with the crystal structures of DFPase.

The optimized geometries of transition states and products were shown in Figure 4 (A: PON1W-Paraoxon, B: PON1M-Paraoxon, C: PON1W-DFP, D: PON1M-DFP). The activated nucleophile W1 attacks the phosphorus center and leads the P–O or P–F bond, broken to form the hydrolysis product. Glu53 helps orientate the W1 by accepting a hydrogen bond for the wild model, but acts as a general base to abstract the second proton from W1 for the mutant models following the corresponding transition state. Both the residues Asn168 and Asn224 help the leaving group leave through hydrogen bonds for models PON1W-Paraoxon, PON1M-Paraoxon, PON1W-DFP, and PON1M-DFP, which is similar to the DFPase [27], while for the PON1W-Paraoxon model, Asn168 and Asn224 keep donating hydrogen bonds to the oxygen atom of phosphate group along the whole reaction path.

The transition states for the nucleophilic attack by the activated nucleophile W1 on the phosphorus center were optimized and confirmed to be the first-order saddle point with an imaginary frequency (99*i* cm^−1^ for the PON1W-Paraoxon model and 108*i* cm^−1^ for the PON1M-Paraoxon model). The key bond distances of the transition states were listed in Table 2. In the mutant model PON1M-Paraoxon (B-TS), the distance of H_a_–O_W1_ is 1.55 Å, 0.05 Å shorter than that in the wild type model A–TS, while the distance of H_b_–O_W1_ is 1.02 Å, 0.01 Å longer than that in A–TS. The key distance of O_W1_–P is 1.66 Å, same as that in A-TS; while the distance of P–O_phosphate_ is 2.21 Å, which is 0.02 Å shorter than that in A-TS.

**Figure 5:**
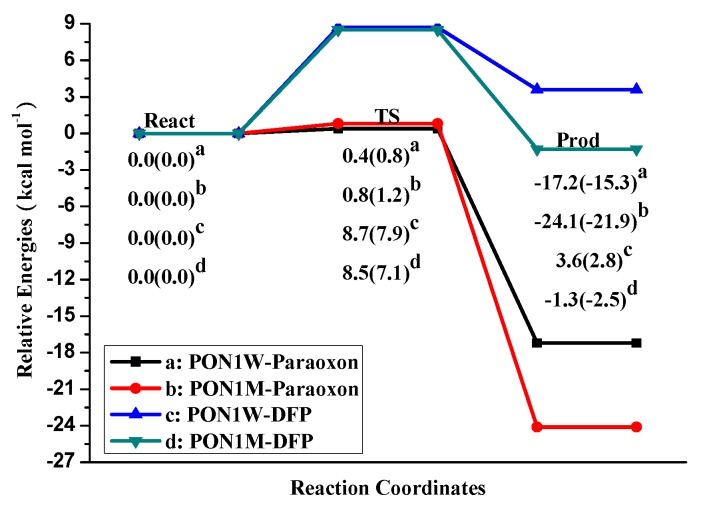
Potential energy profiles of the catalytic reactions for (a) PON1W-Paraoxon, (b) PON1M-Paraoxon, (c) PON1W-DFP, and (d) PON1M-DFP models with and without (in parenthesis) the solvation correction.

The energy profiles for the rate-determining step, the leaving-group elimination (P–O or P–F bond broken) step, were summarized shown in Figure 5. For the PON1W-Paraoxon model, the energy barrier is 0.4 kcal mol^−1^ (0.8 kcal mol^−1^ without the solvation correction). Replacing Asn270 into W2, the energy barrier faintly increases by 0.4 kcal mol^−1^ both with and without the solvation correction. Particularly, compared to the PON1W-Paraoxon product (A-Prod), the stabilities of the PON1M-Paraoxon product (B-Prod) increased ca. 7.0 kcal mol^-1^ relative to the corresponding reactant. Thus, the mutation of Asn270 has little effect on the forward reaction rate of PON hydrolysis, while makes the reverse reaction much harder to take place.

Similar geometry changes were observed in the mutant model PON1M-DFP (Table 2 and Figure 4). The transition states of PON1W-DFP (C-TS) and PON1M-DFP (D-TS) models were optimized and confirmed to be the first-order saddle point with an imaginary frequency 139*i* and 159*i* cm^−1^, respectively. In the PON1M-DFP model (D-TS), the distance H_a_–O_W1_ is 1.64 Å, 0.06 Å shorter than that in the wild type model C-TS. The distance H_b_–O_W1_ is 1.01 Å, 0.01 Å longer than that in C-TS. The key distance of O_W1_–P is 1.65 Å same as that in C-TS; while the distance of P–F is 2.19 Å, which is 0.05 Å shorter than that in C-TS. In the PON1W-DFP model, the P–F bond breaking reaction has a relatively higher energy barrier 8.7 kcal mol^−1^ (7.9 kcal mol^−1^ without the solvation correction), which is consistent with the larger model and basis sets results 6.0 kcal mol^−1^ [27]. Replacing Asn270 into H_2_O, the energy barrier decreases 0.2 kcal mol^−1^ (0.8 kcal mol^−1^ without the solvation correction). However, the mutation makes the forward reaction change from endothermal 3.6 kcal mol^−1^ to exothermal 1.3 kcal mol^−1^ (Figure 5). Thus, replacing Asn270 into H_2_O is essential for DFP hydrolysis, and provides much more driving force for the forward reaction.

From the geometry results (Table 2 and Figure 4), in the mutant model PON1M-Paraoxon, the decreased H_a_–O_W1_ distance and the increased H_b_–-O_W1_ at TS suggest a less nucleophilic hydroxide generated relative to the wild type model. This should be the reason for the faintly increased energy barrier of the paraoxon hydrolysis. The two new hydrogen bonds between Asn168, Asn224, and O_L_ of the leaving group help stabilize the product of the mutant model. In the mutant model PON1M-DFP, the decreased P–F distance at TS suggests a more easily broken P–F bond relative to the wild type model. The much stronger O^2−^ nucleophile makes the reaction easier to take place, due to another proton H_b_ transfer to Glu53, which should also be the reason for the increased exothermic heat of DFP hydrolysis.

In a word, the energy barrier of P–F bond broken in the PON1W-DFP model is higher by about 8 kcal mol^−1^ than that of the P–O bond broken in the PON1W-Paraoxon model. It suggests that the nucleophile water molecule prefers the substrate Paraoxon to DFP in wild type, which agrees with the catalysis characteristics of PON1 [51,52]. Substituting water molecule for Asn270 can weakly reduce the paraoxonase activity and enhance the DFPase activity, and especially, provide essential thermostability for the DFP hydrolysis reaction to occur. However, such enzymatic activity changes mediated by the mutation of Asn270 are too faint to be a decisive factor for the enzymatic properties of PON1 and DFPase. It is consistent with the experimental conclusion that the site-specific saturation mutagenesis indicated Asn270 (among all active-site residues) plays a relatively minor role in catalysis for both paraoxon and TBBL [4].

### 2.2. Effects of Asn270 Mutation on Enzymatic Structure

**Figure 6:**
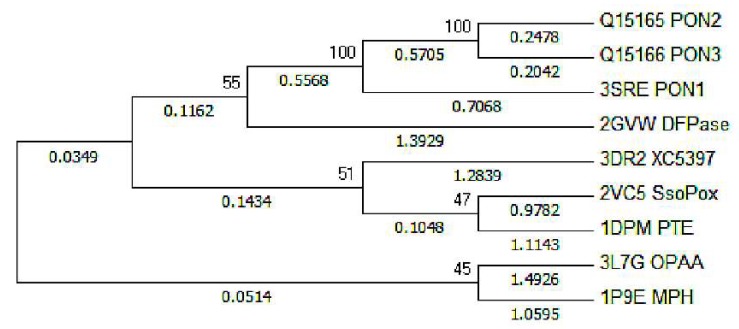
Evolutional relationship of PON1/2/3, DFPase, XC5397, SsoPox, PTE, OPAA, and MPH. The residue sequences are from PDB data or sequence date as shown in sequence names.

Furthermore, taking into account the whole amino acid sequences, a phylogenetic tree of PON1/2/3, DFPase, and XC5397, together with several bimetalloenzymes possessing phosphotriesterase activities, was constructed as shown in Figure 6. Among these enzymes, PON1/2/3, DFPase, and XC5397 adopt mononuclear metal active sites; hyperthermophilic archaeon *Sulfolobus solfataricus* (SsoPox) [53], phosphotriesterase (PTE) [54], organophosphorus acid anhydrolase (OPAA) [55] and methyl parathion hydrolase (MPH) [56] all adopt binuclear metal active sites. The evolutionary relationship by the phylogenetic tree shows that XC5397 adopts a mononuclear metal active site, getting close to SsoPox and PTE, which adopt binuclear metal active site.

**Figure 7:**
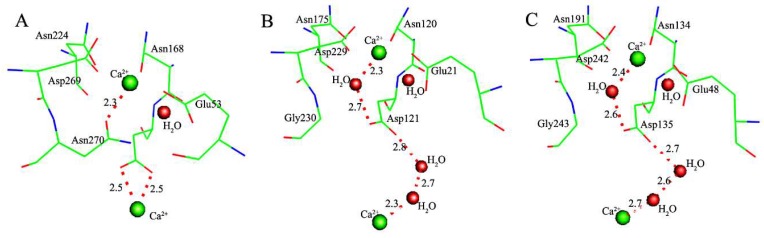
Structures of the catalytic Ca^2+^ ion and the buried structural Ca^2+^ ion in PON1 (PDB code: 3SRE) [15] (**A**), DFPase (PDB code: 2GVW) [18] (**B**), and XC5397 (PDB code: 3DR2) [28] (**C**), respectively. These figures are drawn by the PyMOL [44].

Structure comparisons of evolutionarily related enzymes PON1, DFPase, and XC5397 show that the residue ligand Asn270 in the active site of PON1 is mutated into Gly230 in DFPase (Gly243 in XC5397), of which the coordination of catalytic Ca^2+^ ion was completed by a water molecule (W2) (Figure 7). It turns out that the catalytic Ca^2+^ ion indirectly connects the buried structural Ca^2+^ ion via a series of hydrogen bonds involving Asp121 in DFPase (Asp135 in XC5397) and three water molecules (including two coordinated water molecules). Significantly, such connections between both Ca^2+^ ions are highly consistent in DFPase and XC5397.

Co-evolving with the mutation of Asn270, the catalytic Ca^2+^ ion indirectly connects the buried structural Ca^2+^ ion via a series of hydrogen bonds involving Asp121 and water molecules in DFPase (Figure 7). Together with the evolutionary relationship shown by the phylogenetic tree (Figure 6), it implies an evolutionary transition from mono- to dinuclear catalytic centers, which is also suggested by Moshe Ben-David et al. [4,15,57]. Such connections of Ca^2+^ ions can reduce the plasticity of catalytic structure, and weaken the promiscuous binding of ligands and the catalytic promiscuity. It can be interpreted that PON1, with higher plasticity of catalytic structure, can accommodate promiscuous binding of ligands, such as relatively large substrate paraoxon, while DFPase, with lower plasticity of catalytic structure, prefers relatively small substrate DFP instead of accommodating promiscuous substrates. It also can be concluded that Asn270 in PON1 plays a minor role on enzyme catalytic reaction, but plays a key role on the plasticity of catalytic structure. This conclusion is supported by our previous studies that a charged residue, such as aspartate or glutamate in the active site, plays a much more considerable role on catalytic reaction than a neutral residue [8].

## 3. Methods 

All calculations in this study were performed using DFT method with the B3LYP function [58,59,60]. The 6-31G(d, p) basis set was used for the carbon, hydrogen, oxygen, nitrogen, and calcium atoms, the 6-311+G(d) basis set was used for phosphorous atom. At the same level, the frequency calculations were performed to obtain zero-point energies (ZPE) and to confirm the nature of the stationary points and transition states along the reaction profiles. Intrinsic reaction coordinate (IRC) calculations were also explored to make sure that the transition state connects the corresponding reactant and the product.

The polarization effects of the enzyme environment were evaluated by performing single-point calculations on the optimized structures at the same theory level as the geometry optimizations using the conductor-like polarizable continuum model (CPCM) method [61,62,63,64]. The dielectric constants were set to four and eighty, which are usually used in modeling protein surroundings and the aqueous solution, respectively. The barrier discrepancies are only 0.1 kcal mol^−1^ with the constants 4 and 80; here, we just present the results with *ε* = 4. All calculations were performed with the Gaussian 03 program package [65].

The topological tree was constructed using Neighbour-Joining in MEGA 6.06 [66]. Sequence alignment was performed with Clustal W [67].

## 4. Conclusions

In the present work, we have studied the role of Asn270 played in PON1 on hydrolyzing paraoxon and DFP using the popular density functional method B3LYP. The wild type and mutant models were designed, based on the difference of the active sites between PON1 and DFPase (the first shell residue Asn270 replaced by a water molecule). The results suggests the mutation of Asn270 has little effect on the enzyme activity in kinetics, while provides more efficient in thermodynamics, which is essential for DFP hydrolysis.

Furthermore, the structures of evolutionarily related enzymes PON1, DFPase, and XC5397 were compared, which proposes the mutation of Asn270 leads to that the catalytic Ca^2+^ ion indirectly connects the buried structural Ca^2+^ ion via hydrogen bonds in DFPase. Such connections between Ca^2+^ ions can reduce the plasticity of enzymatic structure, and possibly alter the substrate preference from paraoxon to DFP, which also implys an evolutionary transition from mono- to dinuclear catalytic centers.

## Figures and Tables

**Table 1:** Coordination bond lengths of the optimized geometries for the Active-site-W, Active-site-M, PON1W-Paraoxon, PON1M-Paraoxon, PON1W-DFP, and PON1M-DFP models. “Δ” is the difference relative to the Active-site-W model. Distances are in angstroms.

Coordination Bonds	Active-Site-W	Active-Site-M/Δ	PON1W-Paraoxon/Δ	PON1M-Paraoxon/Δ’	PON1W-DFP/Δ	PON1M-DFP/Δ’
**Ca-O_Glu53_**	2.51	2.47/−0.04	2.45/−0.06	2.37/−0.14	2.46/−0.05	2.39/−0.12
**Ca-O_Asn168_**	2.73	2.67/−0.06	2.61/−0.12	2.52/−0.21	2.64/−0.09	2.56/−0.17
**Ca-O_Asn224_**	2.40	2.38/−0.02	2.42/0.02	2.46/0.06	2.43/0.03	2.48/0.08
**Ca-O_Asp269_**	2.49	2.51/0.02	2.52/0.03	2.64/0.15	2.51/0.02	2.62/0.13
**Ca-O_Asn270_**	2.41	/	2.42/0.01	/	2.43/0.02	/

**Table 2:** Important bond distances of TS in the PON1W-Paraoxon, PON1M-Paraoxon, PON1W-DFP, and PON1M-DFP models. “Δ” is the difference relative to the wild type models. Distances are in angstroms.

TS Bonds	PON1W-Paraoxon	PON1M-Paraoxon/Δ	PON1W-DFP	PON1M-DFP/Δ
**H_a_-O_W1_**	1.60	1.55/−0.05	1.70	1.64/−0.06
**H_b_-O_W1_**	1.01	1.02/0.01	1.00	1.01/0.01
**O_W1_-P**	1.66	1.66/0.00	1.65	1.65/0.00
**P-O/F**	2.23	2.21/−0.02	2.24	2.19/−0.05
